# Dasymetric population mapping based on US census data and 30-m gridded estimates of impervious surface

**DOI:** 10.1038/s41597-022-01603-z

**Published:** 2022-08-27

**Authors:** Rachel H. Swanwick, Quentin D. Read, Steven M. Guinn, Matthew A. Williamson, Kelly L. Hondula, Andrew J. Elmore

**Affiliations:** 1 National Socio-Environmental Synthesis Center, Annapolis, MD 21401 USA; 2 Rubenstein School of Environment and Natural Resources, University of Vermont, Burlington, VT 05405 USA; 3 Agricultural Research Service, United States Department of Agriculture, Raleigh, NC 27606 USA; 4 Integration and Application Network, University of Maryland Center for Environmental Science, Annapolis, MD 21403 USA; 5 Appalachian Laboratory, University of Maryland Center for Environmental Science, Frostburg, MD 21532 USA; 6 Human-Environment Systems, Boise State University, Boise, ID 83725 USA; 7 Center for Global Discovery and Conservation Science, Arizona State University, Tempe, AZ 85287 USA

**Keywords:** Socioeconomic scenarios, Environmental impact, Natural hazards

## Abstract

Assessment of socio-environmental problems and the search for solutions often require intersecting geospatial data on environmental factors and human population densities. In the United States, Census data is the most common source for information on population. However, timely acquisition of such data at sufficient spatial resolution can be problematic, especially in cases where the analysis area spans urban-rural gradients. With this data release, we provide a 30-m resolution population estimate for the contiguous United States. The workflow dasymetrically distributes Census block level population estimates across all non-transportation impervious surfaces within each Census block. The methodology is updatable using the most recent Census data and remote sensing-based observations of impervious surface area. The dataset, known as the U.G.L.I (updatable gridded lightweight impervious) population dataset, compares favorably against other population data sources, and provides a useful balance between resolution and complexity.

## Abstract (summary)

**Table:** 

Measurement(s)	Population Density
Technology Type(s)	satellite imaging
Sample Characteristic - Organism	Homo sapiens
Sample Characteristic - Environment	populated place
Sample Characteristic - Location	contiguous United States of America

## Background & Summary

There is growing awareness that solutions to pressing challenges in environmental science require characterizing interactions and feedbacks between social and natural systems^1,2^. Socio-environmental systems (SES) are highly complex and key to the assessment of their dynamics, including the provisioning of ecosystem services and risks posed by environmental hazards and public health outcomes, is linking people to the environment with which they interact^3^. Therefore, SES research requires a detailed understanding of where people live in relation to environmental factors. Whereas, environmental systems data is increasingly available at high levels of spatio-temporal resolution through advanced remote sensing technologies, the provision of population data at a similar resolution has been more challenging^4^. This data release answers the need for spatially resolved population distribution estimates that are integral to informing research, policy and management decisions across a range of SES challenges^5^.

Population data only attains high accuracy when aggregated spatially. In the United States (U.S.), population data is provided by the U.S. Census Bureau through the decennial census and American Community Survey, and many other countries have similar programs. The U.S. Census Bureau aggregates population estimates to areal units such as counties or census blocks, which vary in size based on population density, but do not natively support high-resolution gridded population estimates. Typical census data is aggregated to ensure that each spatial unit has a minimum population size. Although this aggregation facilitates sampling design and ensures that responses remain anonymous, it can also lead to inferential problems when attempting to analyze SES at a fine spatial grain^6,7^. For example, census units in urban areas are relatively small and populations are evenly distributed, whereas units in rural areas are larger and populations are irregularly distributed^8^. This results in the modifiable areal unit problem, that makes census data inadequate to project population density and distribution particularly in rural communities^7,9^. It also suggests that locational accuracy of human populations is lower in rural communities than in more densely populated areas. Nevertheless, simple areal weighting within aggregation units is a practical approach to generating gridded population estimates, and it is commonly employed when coarse resolution population estimates over large spatial extents are the goal^5^.

Many studies have discussed approaches for generating and using gridded population data to overcome the pitfalls of aggregated national census data^5,10,11^. These approaches are based on the observation that geographic features indicating the built and natural environment are strong predictors of population distributions at fine spatial scales^12^. Once maps of these geographic features are in hand, dasymetric mapping is used to disaggregate coarse resolution population estimates (e.g. national census) to produce a finer resolution estimate of population distribution^5,13^. The inclusion of a high-resolution product allows for a more in-depth analysis of landscape features (e.g. residential units, roads, water bodies, protected areas) that help to define how census population estimates are distributed within units of aggregation. Methods used in dasymetric mapping are varied and depend on the number of geographic datasets employed and the thematic resolution of input data (e.g., binary, multi-class, or continuous)^5^. The most intuitive approach is to apply empirical relationships defining population weights, ranging from binary to continuous, for each geographic feature. More complex approaches apply multivariate statistics or machine learning to relate a large selection of geographic variables to population observations^14^. Each of these approaches present trade-offs in complexity, required data, and ability to easily update gridded results as new data become available. Trade-offs also exist with interpretability as methods based on artificial intelligence provide little information on how the geographic predictors are related to population^15^.

Despite this broad body of work, most products either cover a specific geography or are provided at low resolution. Furthermore, there remains a need for high resolution population estimates that are based on reliable maps of the built environment that can be updated easily as new population data become available. This data release of the U.G.L.I. (updatable gridded lightweight impervious) population dataset fills the described need through the production of a 30-m gridded population map for the contiguous United States (CONUS) that provides a novel level of customization, repeatability and updateability compared to other readily available dasymetric mapping data products. To generate the map, we use publicly available population data from the U.S. Census Bureau and impervious surface area data from the National Land Cover Database (NLCD)^16^. The method is relatively simple, and can therefore be updated as new population and land-cover data become available. Finally, the data provide the same population estimates as Census data when aggregated to the block level or above but with greater accuracy attributed to distribution of these population estimates.

## Methods

### Overview

**Fig. 1:**
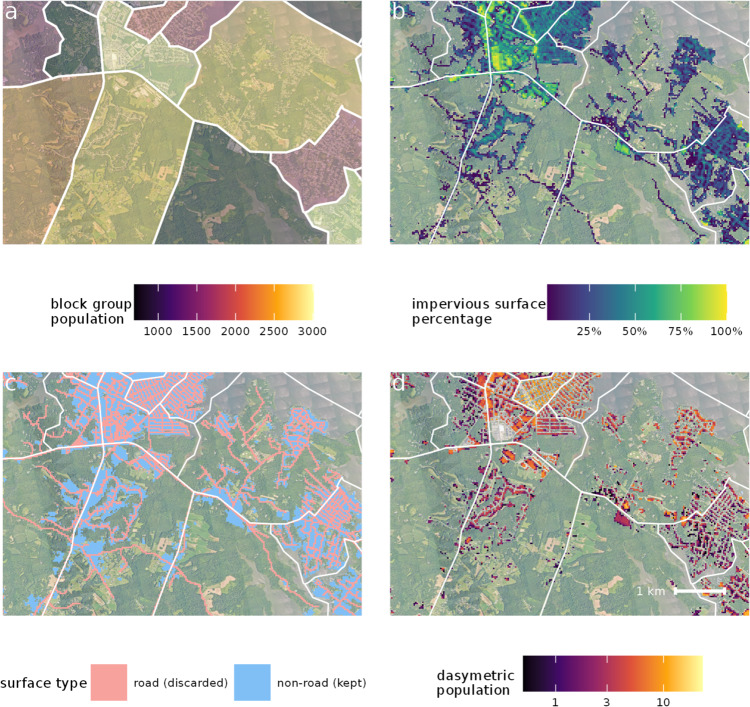
Elements of the dasymetric population mapping workflow. (**a**) Block group population estimates for south-central Anne Arundel County, Maryland (boundaries between block groups shown in white). (**b**) Impervious surface area (30 m) from the NLCD. (**c**) Impervious surface classification, showing roads and non-road areas. (**d**) The final population map, with population distributed across non-road impervious surface area and all Census blocks with zero population removed.

In the U.S., the county is the only hierarchical unit that never splits a block group. The county is also a convenient size to allocate to a single computational unit, thus optimizing parallel processing. Therefore, the county (or county equivalent) was selected as the processing unit for dasymetric population mapping. First, for each county, we obtained the five-year population estimates from the 2016 American Community Survey (ACS^17^) for each Census block group (Fig. 1a). Using imperviousness data from the National Land Cover Database (NLCD^18^), we assigned a population of zero to all 30-m pixels with no impervious surface area (Fig. 1b), pixels containing roads (using the NLCD Impervious Surface Descriptor product; Fig. 1c), and pixels in Census blocks with zero population. We then apportioned the population of each remaining block group among the remaining pixels based on the total amount of impervious surface area contained within each pixel. This resulted in a 30-m resolution dasymetric population map (Fig. 1d). When re-aggregated to the Census block group or larger scale, U.G.L.I. reports the identical population as reported by the U.S. Census input data.

### Data sources

Our dasymetric population estimation method uses data provided by the U.S. Census Bureau and the Multi-Resolution Land Characteristics (MRLC) Consortium (Table 1). The Census data products are the 5-year population estimates at the U.S. Census block group level derived from the 2016 ACS and population counts at the U.S. Census block level derived from the 2010 decennial Census. We obtained all Census data from the U.S. Census Bureau API. We also obtained the geographic boundaries of blocks and block groups from the Census API. The MRLC data products used are the NLCD Percent Developed Imperviousness product for 2016 for the CONUS, and the NLCD Impervious Surface Descriptor product for 2016 for the CONUS. We used the April 2019 data release for both NLCD data products. A more recent release is now available from MRLC, but we include the NLCD layers from 2016 in this data release for reproducibility.

**Table 1:** Data sources used in U.G.L.I dasymetric population mapping.

Data product	Provider	Year	Resolution	Source
Block group 5-year population estimates, American Community Survey	U.S. Census Bureau	2016	Census block group	^17^
Block population counts, decennial Census	U.S. Census Bureau	2010	Census block	^34^
Census block and block group geographic boundaries	U.S. Census Bureau	2016	Census block	^35^
Impervious surface area	MRLC	2016	30 m	^36^
Impervious surface descriptor	MRLC	2016	30 m	^37^

### Spatial processing

After obtaining Census Bureau data, we projected block and block group geographies to the Albers equal-area projection used by NLCD. Next, we clipped the NLCD impervious surface percentage and descriptor rasters to the county area (the spatial union of all block groups in the county). All subsequent methodological steps were applied at the county level for all counties or county equivalents in the CONUS.

### Using impervious surface to identify potentially populated areas

Data layers representing impervious surface area, the impervious surface area descriptor, and 2010 Census block population estimates, were used to identify pixels where impervious surfaces could be assumed to represent locations where people live. We first set all impervious surface pixels with a value less than or equal to 0.01 to 0 (less than 1% of the pixel’s surface area is impervious). Secondly, all pixels in Census blocks with zero population in the 2010 decennial Census were set to 0 regardless of what impervious surfaces they contained. It was assumed that impervious surfaces within 2010 Census blocks with zero population represent non-domestic impervious surfaces, such as commercial and industrial land uses, which are important to remove before dasymetric operations^19^. The 2010 Census blocks were used for this operation because these data represent the finest grain Census population data available. Finally, the impervious surface descriptor, which classifies all impervious surfaces by source, was used to set all pixels classified as primary, secondary, and tertiary urban roads to 0 (Fig. 1). This process retained non-road impervious surfaces. The NLCD impervious data layers include input from the Microsoft US Building Footprint dataset, which increases overall accuracy of these data to above 90% and is particularly important for including impervious area over buildings with small footprints^20^. The resulting modified impervious surface area layer contained the fraction of impervious surface area in all pixels where it was valid to assume that population was greater than zero, and zero elsewhere. Considering the steps taken to remove non-residential impervious surfaces, this assumption was in line with past work examining the relationship between impervious surface and population^19,21^.

### Dasymetric population density estimation

**Fig. 2:**
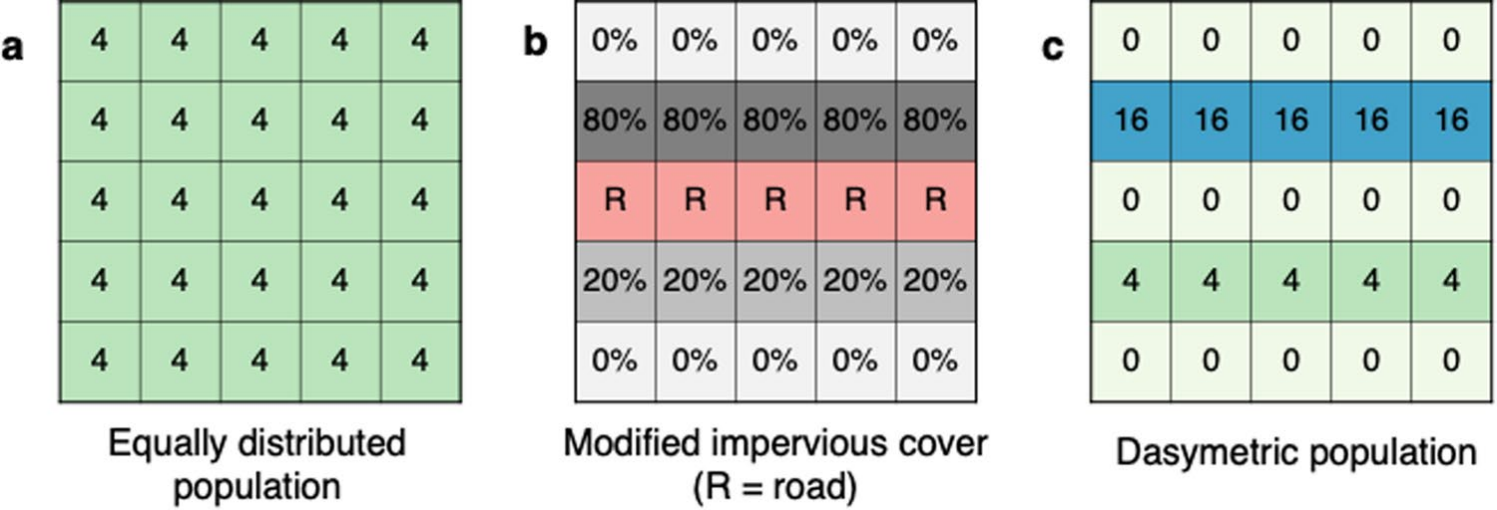
Per pixel distribution of a hypothetical 100-person census block group. (**a**) shows population counts assuming an equal distribution (e.g. census data). (**b**) shows the modified impervious cover layer used to calculate (**c**), the dasymetric population in each pixel based on the proportion of block group residential impervious cover (e.g. U.G.L.I population dataset).

To perform the dasymetric population estimation for each county, we used the 2016 Census block group population estimates as opposed to the 2010 Census block population counts. The block group is a larger level of aggregation than the block, but is provided by the U.S. Census more frequently and is also coincident with the 2016 NLCD impervious data layers. Future users of the provided code could choose to use other population data. For each county, we rasterized the 2016 Census block group population data to match the resolution and extent of the modified impervious surface area layer (i.e., those pixels remaining after the procedures described above). For each non-zero pixel in the modified impervious surface area layer, we assigned population based on the proportion of the modified impervious surface area within the block group contained in that pixel (Fig. 2). The result contained a population estimate for each pixel that was scaled to the total population of the block group, thus the sum of all pixels in a block group exactly equaled the block group population.

### Correction for anomalous counties

Because we used Census Bureau data products from both 2010 and 2016, there are two inconsistencies. Between 2010 and 2016, Shannon County, South Dakota was renamed Oglala Lakota County, resulting in a new FIPS code assignment, and the independent city of Bedford, Virginia merged with its surrounding county Bedford County. We corrected these anomalies as follows. In the case of Oglala Lakota County, we used the 2016 block group population estimates for Oglala Lakota County and the 2010 block population counts for Shannon County. In the case of Bedford County, we used the 2016 block group population estimates for Bedford County (which includes Bedford City) and we merged the 2010 block population counts for Bedford City and Bedford County.

### Computational infrastructure

We ran the code in R 4.0.3 and GDAL 2.2.2 on a Slurm cluster running the Linux-based operating system Ubuntu 16.04. The code ran in approximately 24 hours across 7 cluster nodes, each with 8 processor cores.

## Data Records

U.G.L.I data products can be accessed at Figshare in GeoTiff format^22^. The data are 30 m in resolution, are subset by county, and projected in the Albers Equal Area projection. The value of each pixel in the data set is the estimated population in the 900 m^2^ area. Each file name includes a county code^23^ of the form “XX-YYY”, where XX designates the state and YYY designates the county.

## Technical Validation

To demonstrate the fitness for use^5^ of the U.G.L.I. dataset, we explored how our method to dasymetrically estimate population distribution might affect policy-relevant inference compared to previously published estimates. We investigated two key environmental hazards: wildfire and coastal flooding events. In each case, raster data products are available and have been recently used to assess wildfire^24^ and flood hazard risk^25^. The U.S. Forest Service produced a map of wildfire hazard potential (WHP) for the contiguous United States at 270-meter pixel resolution, with five risk categories^26^. The U.S. Federal Emergency Management Agency (FEMA) released flood risk data products for many U.S. counties, including water surface elevation (WSE) for a 1% flood event (expected to occur once every 100 years). The WSE product is provided at 10-meter pixel resolution^27^. Both of these products define a spatial area at risk and not the number of people in that area.

For each of the two hazard categories, we compared estimation methods from four different sources: (1) U.G.L.I. (i.e., the present study), (2) the U.S. Environmental Protection Agency (EPA^28^), (3) Microsoft^29^, and (4) Facebook^9^ (Table 2). In addition, we compared all the methods to a fifth baseline method: assuming that individuals are evenly distributed across the entire geographical area of each Census block group^30^. Note that each of these data sets uses different methods. While they are all freely available data sets, only the EPA product could be updated by a third party. The EPA product uses the EPA’s Intelligent Dasymetric Mapping toolbox, which combines US Census, and data on land cover and topography^28^. The Microsoft product uses the footprints of buildings and their sizes to disaggregate Census block group population estimates. And the Facebook dataset uses artificial intelligence algorithms of largely unknown structure. We chose these population sources because they are all available for the U.S. and are provided at roughly the same spatial resolution as U.G.L.I. The final method, that evenly distributes population within each block group, was used as reference against which all methods were compared.

**Table 2:** Data sources for technical validation.

Data product	Provider	Data year	Resolution	Coverage	Source URL
Wildfire hazard potential (WHP)	U.S. Forest Service	2020	270 m	CONUS	^26^
Water surface elevation (WSE) for 1% flood event	U.S. FEMA	varies	10 m	County	^27^
Dasymetric population raster	U.S. EPA	2016	30 m	CONUS	^31^
Dasymetric population raster	Huang *et al*. 2021 Microsoft	2017	100 m	CONUS	^29^
Dasymetric population raster	Facebook	2019	30 m	Global	^15^

### Selection of counties for case study

For the wildfire case study, we took a random sample, stratified by population, of 15 counties in the eleven western states of the contiguous U.S. (WA, OR, CA, ID, NV, MT, WY, CO, UT, AZ, NM), sampling three counties in each population quintile. We chose five counties to display in the final visualization that have sufficient spatial variation in wildfire risk to differentiate between the population methods. For the flood case study, we took a population-stratified random sample from all counties bordering a coastline in the contiguous U.S. However, FEMA does not provide flood risk data products for all counties, so we continued sampling until we had ten counties where flood risk data products were available. Again, we chose five counties to display examples that best illustrate the differences between the population methods.

### Data sources

We obtained the wildfire hazard potential raster product for the entire contiguous United States, and the 1% flood event water surface elevation product for each of the selected counties. We obtained the gridded population estimates for the three comparison methods as raster layers covering the contiguous United States (Table 2). Finally, we used the previously obtained population estimates for 2016 from the American Community Survey for each of the counties chosen for the case study, as well as the boundaries of each block group as a polygon layer (Table 1).

### Initial raster processing

We clipped the wildfire hazard potential raster layer and the population raster layers (U.S. EPA^31^, Microsoft^29^, and Facebook^15^) to the extent of each county in the case study. The water surface elevation rasters were already provided at the single county level. For simplicity, we converted both environmental raster layers to binary form (i.e., at risk and not at risk). For the wildfire layer, we treated all pixels in the medium, high, and very high risk categories as being at risk, and the remainder as not at risk. For the flooding layer, we treated all pixels with water surface elevation >0 as being at risk. Next, we converted the wildfire and flooding rasters to polygons by merging all adjacent pixels with the same value into a polygon. Finally, we transformed these polygon layers into the coordinate reference system of each of the population rasters.

### Estimating population totals in each risk category

We overlaid the feature layers representing wildfire and flood risk onto the population raster layers for each dasymetric estimation method. For each wildfire and flood polygon, we summed the population across all pixels contained within that polygon, then calculated the grand totals for each risk category in each county.

For the block group population polygons, we calculated the areas of intersection of each block group polygon within each wildfire or flood polygon. We multiplied the population total of the block group polygon by the proportional area of overlap between each environmental risk polygon to yield the population total at risk within each block group, then calculated totals for each county.

**Fig. 3:**
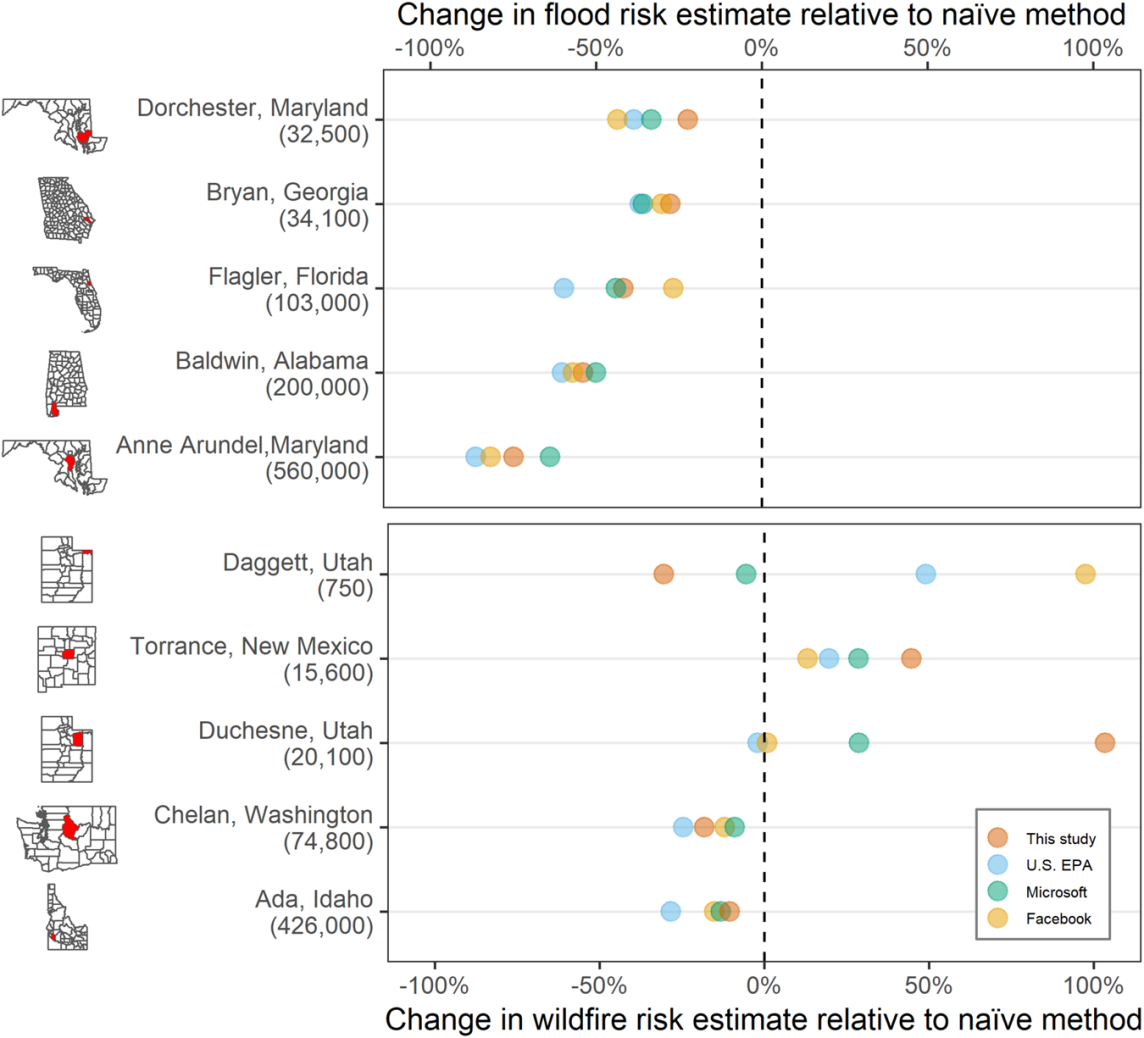
A comparison of the estimated population at risk due to environmental hazards using 4 different population maps, each expressed as the percent difference relative to a naïve method that assumes an even distribution of population across each Census block group. We examined two case studies: flooding risk (top) and wildfire risk (bottom). Data for five counties are compared in each case study, with their total 2016 American Community Survey population estimates given in parentheses.

The results of this technical validation confirm that our dataset provides a highly comparable assessment of population estimates at risk from flooding and wildfire using dasymetric mapping (Fig. 3). In the case of flooding, all four population estimates were lower than the naïve method. While the naïve method places people evenly across Census block groups, each of these methods correctly identifies floodplains as locations containing fewer people than upland areas. The consistency observed here demonstrates that there are no logic errors in the U.G.L.I. processing and that it produces data that on a first order is comparable to other methods. In the case of fire risk, we observed greater variation between population data sets. In three of the five counties, the difference was small. One of the final two counties has a very small population (750 people), which likely causes greater variance between models. While there is no obvious reason why the population methods diverge in some counties, we can assume it has something to do with the model assumptions used in each case. The data is not available to know which method produces the most accurate result.

Our dataset offers several advantages as compared to other dasymetric population data packages, and contains many of the same uncertainties. Because the code provided with this data release can be updated as new data becomes available, it can be customized to include different data sources and has a high level of reproducibility. Further, it was produced in a very intuitive way that should aid communication to stakeholders. Future improvements might include the inclusion of additional factors that influence where people live^32^. Uncertainty in population estimates provided by U.G.L.I. likely stem from the underlying data (e.g., impervious surface estimates) and assumptions (e.g. distribution of population across impervious surfaces). The NLCD impervious surface data has stated accuracy over 90%^20^, which is excellent, and we would expect the accuracy of U.G.L.I. to improve as the NLCD improves. The ability to map population at a grain of 30 m is critical for understanding populations at risk of natural disasters, but also identifying populations that benefit from ecosystem services such as water and trail access, forest canopies, and protected land. As more data with these qualities becomes available, we expect a greater diversity in uses to become apparent.

## Data Availability

All R code required to reproduce the U.G.L.I data products and validation analysis is available at the following 10.5281/zenodo.5750665^33^.
